# Common Features Between the Proteomes of Floral and Extrafloral Nectar From the Castor Plant (*Ricinus Communis*) and the Proteomes of Exudates From Carnivorous Plants

**DOI:** 10.3389/fpls.2018.00549

**Published:** 2018-04-27

**Authors:** Fábio C. S. Nogueira, Andreza R. B. Farias, Fabiano M. Teixeira, Gilberto B. Domont, Francisco A. P. Campos

**Affiliations:** ^1^Proteomics Unit, PPGBq, Department of Biochemistry, Institute of Chemistry, Federal University of Rio de Janeiro, Rio de Janeiro, Brazil; ^2^Laboratory of Proteomics, LADETEC, Institute of Chemistry, Federal University of Rio de Janeiro, Rio de Janeiro, Brazil; ^3^Department of Agricultural Sciences, Federal University of Ceará, Fortaleza, Brazil; ^4^Department of Biochemistry and Molecular Biology, Federal University of Ceará, Fortaleza, Brazil

**Keywords:** floral nectar, extrafloral nectar, carnivorous plants, *Ricinus communis*, proteomics

## Abstract

Label-free quantitative proteome analysis of extrafloral (EFN) and floral nectar (FN) from castor (*Ricinus communis*) plants resulted in the identification of 72 and 37 proteins, respectively. Thirty proteins were differentially accumulated between EFN and FN, and 24 of these were more abundant in the EFN. In addition to proteins involved in maintaining the nectar pathogen free such as chitinases and glucan 1,3-beta-glucosidase, both proteomes share an array of peptidases, lipases, carbohydrases, and nucleases. A total of 39 of the identified proteins, comprising different classes of hydrolases, were found to have biochemical matching partners in the exudates of at least five genera of carnivorous plants, indicating the EFN and FN possess a potential to digest biological material from microbial, animal or plant origin equivalent to the exudates of carnivorous plants.

## Introduction

Nectar is an energy rich substance secreted by glands situated at the base of flowers (floral nectar, FN) or in other parts such as leaves, stems, rachis, etc. (extrafloral nectar, EFN) (Shah et al., 2016). While FN attracts pollinating insects, EFN attracts aggressive ants and other mutualists, which in turn provide antiherbivore protection (Marazzi et al., 2013). Although these functional aspects are widely recognized (Roy et al., 2017), the dynamic of the relation FN/pollinators and EFN/mutualists is poorly known, especially the biochemical properties of the nectar which play a role in the attraction of particular pollinator/mutualists. Additionally, little is also known about the biochemical machinery involved in the secretion of nectar (Heil, 2015), and even less on the proteins responsible for maintaining these carbohydrate rich energy sources free of pathogens (Park and Thornburg, 2009; Heil, 2015; Roy et al., 2017). Up to now, only a limited number of studies have presented data on the proteomes of EFN and FN (Orona-Tamayo et al., 2013; Seo et al., 2013; Zhou et al., 2016). These studies demonstrated the worth of establishing the complete proteomes of EFN and FN to acquire a better understanding of the preference of certain mutualists for a particular type of EFN or FN and paved the way for establishing that proteins in nectars have roles which go beyond helping in keeping the nectar pathogen free.

The notion that the proteome of EFN is larger than FN is well established (Coulter et al., 2012) but it has not yet been tested directly. Likewise, it is still not known whether nectars from different sources possess a common set of proteins with the general role of keeping it pathogen free and a variable number of proteins conferring to a certain EFN or FN properties underlying its acceptance/rejection by mutualist animals. Last of all, the possibility that EFN and FN are involved in aspects of plant biology other than pollination and defense have not been investigated so far.

In order to address these questions, we have performed a label-free quantitative proteome analysis of EFN and FN from the castor plant (*Ricinus communis*) using nectar collected from plants grown under similar temporal and environmental conditions. Our analysis provides evidence for the presence in EFN and FN of a wide array of hydrolases (peptidases, carbohydrases, lipases, and nucleases) and a number of proteins related to the dismantling of the cell wall of plants and fungi. Additionally, we show that a sizable fraction of the proteins from EFN and FN have counterparts in the proteomes of the exudates of carnivorous plants.

## Materials and methods

### Acquisition of floral (FN) and extrafloral (EFN) nectar

Plants were grown under irrigation, in the experimental field of the Agronomy School, Federal University of Ceará, Fortaleza, Brazil. Nectar collection was performed daily from 6 to 8 a.m. (1–3 h after sunrise), by the use of a handmade glass syringe, totalizing four and three biological samples for the FN and EFN, respectively. The material collected was immediately centrifuged (10,000 g), and sterile-filtered and kept at −20°C until used.

### Protein precipitation and trypsin digestion

Collected FN and EFN were submitted to protein precipitation using cold acetone with 10% TCA as described (Vasconcelos et al., 2005). Precipitated proteins from both FN and EFN were solubilized in 7 M urea, 2 M thiourea. An aliquot was used to determine protein concentration by the Qubit Protein Assay Kit (Qubit® 2.0 Fluorometer, Thermo Scientific) according to the manufacturer's instructions. For protein digestion, 50 μg of proteins of each sample was reduced with dithiothreitol at a final concentration of 10 mM for 1 h at 30°C, followed by iodoacetamide alkylation at 40 mM final concentration for 30 min at room temperature in the dark. Samples were diluted with 50 mM ammonium bicarbonate to 1 M urea concentration and after trypsin addition (1:50, w/w, Sequencing Grade Modified Trypsin, V5111, Promega), solutions were incubated at 35°C for 18 h. Tryptic hydrolysis was stopped with TFA at 0.1% final concentration. After digestion peptides were concentrated and desalted by custom-made chromatographic Poros 50 R2 (PerSeptive Biosystems) reverse phase tip-columns and dried on vacuum concentrator (Thermo Scientific) (Gobom et al., 1999).

### nLC-MS analysis

Peptides resuspended in 0.1% formic acid were quantified by the Qubit Protein Assay Kit. MS analysis was performed in triplicates for each biological replicate from FN and EFN samples in a nano-LC EASY-II coupled to an LTQ-Orbitrap Velos mass spectrometer (Thermo Scientific). Two μg of peptides were loaded in a precolumn (2 cm length, 100 μm I.D., packed in-house with ReproSil-Pur C18-AQ 5 μm resin–Dr. Maisch GmbH HPLC) and fractionated in a New Objective PicoFrit® Column (25 cm length, 75 μm I.D., packed in-house with ReproSil-Pur C18-AQ 3 μm resin–Dr. Maisch GmbH HPLC). Peptides were eluted using a gradient from 95% phase A (0.1% formic acid, 5% acetonitrile) to 40% phase B (0.1% formic acid, 95% acetonitrile) for 107 min, 40–95% phase B for 5 min and 95% B for 8 min (total of 120 min at a flow rate of 200 nL/min). After each run, the column was washed with phase B and re-equilibrated with phase A. m/z spectra were acquired in a positive mode applying data-dependent automatic MS and MS/MS acquisition. MS scans (m/z 350–2,000) in the Orbitrap mass analyzer at resolution 30,000 (at m/z 400), 1 × 10^6^ AGC and 500 ms maximum ion injection time, were followed by HCD MS/MS of the 10 most intense multiply charged ions in the Orbitrap at 10,000 signal threshold, resolution 7,500 (at m/z 400), 50,000 AGC, 300 ms maximum ion injection time, m/z 2.5 isolation width, 10 ms activation time at 30 normalized collision energy and dynamic exclusion enabled for 30 s with a repeat count of 1.

### Database search

Raw data were inspected in Xcalibur v.2.1 (Thermo Scientific). Database searches were performed using Proteome Discoverer 2.1 (Thermo Scientific) using Sequest^TM^ algorithm against *Ricinus communis* database downloaded from Uniprot database March 2017. The searches were performed with the following parameters: MS accuracy 10 ppm, MS/MS accuracy 0.1 Da, trypsin digestion with two missed cleavage allowed, fixed carbamidomethyl modification of cysteine and variable modification of oxidized methionine and acetyl at protein N-terminus. Protein groups and, numbers of peptides were estimated using Proteome Discoverer using false discovery rates around 1% at protein and peptide level and peptide rank. Three technical replicates were obtained for each FN and EFN samples, constituted by four and three biological replicates, respectively. Proteins were considered identified when present in at least two technical replicates for each biological replicate and in at least two biological replicates for each nectar sample. Proteins were filtered by FDR less than 1% and the presence of at least one unique peptide.

### Data analysis

Quantification was estimated using the workflow node Precursor Ions Area Detector in Proteome Discoverer. The peak area average of the most abundant distinct peptides of each protein was used for its relative quantification. Proteins with peak area averages present in at least two technical and two biological replicates were used to generate the list of proteins quantified. Normalization was executed using the total peptide amount where the total sum of the abundance values over all peptides identified within a file is used to correct the abundance in all files. Afterward, the values for the FN and EFN runs were merged and the total median was determined. A ratio of each protein between FN and EFN samples was measured and a *t*-test was performed to evaluate significant differences.

Proteins identified with the database description unclear or as “putative uncharacterized protein” were submitted to manual Blastp in Uniprot (http://www.uniprot.org/blast/) and NCBI (https://blast.ncbi.nlm.nih.gov) websites. Proteins with high identity were selected for the identification of uncharacterized proteins. The subcellular localization was predicted by TargetP (http://www.cbs.dtu.dk/services/TargetP/) (Emanuelsson et al., 2000) and the Top Hit Domains present in the identified proteins was evaluated by PFAM Batch sequence search (https://pfam.xfam.org/search).

## Results

Label-free quantitative proteomics was employed to characterize the proteins present in the extrafloral (EFN) and floral nectars (FN) of castor plants (*Ricinus communis*). The proteomes of EFN and FN are populated by 72 and 37 proteins, respectively (Table 1, Table S1). For FN, 19, 11 and seven proteins were present in two, three, and four biological replicates, respectively. In the EFN, 62 and 10 proteins appeared in two and three biological replicates, respectively. From these, 30 are shared by both nectar types while 7 and 42 are restricted to FN and EFN respectively. As assessed by the TargetP software, 70% of the identified proteins have an N-terminal signal peptide for the secretory pathway (Table S1). Among the proteins unique to the EFN, 20 of them have biochemical counterparts in the FN (Table 1); however, the EFN proteome has a greater complexity in terms of diversity of kinds of enzymatic activities. Beta-fructofuranosidase (B9R9R9) an enzyme known to balance sucrose levels in the extrafloral nectar of several species is among the proteins unique to the EFN.

**Table 1:** Functional classes of proteins identified in EFN and FN proteomes from castor plants (*Ricinus communis*), and the genera of carnivorous plants in which counterpart proteins were identified.

**Sample**	**Accession**	**Description**	**Genus**	**References**
**PEPTIDASES**				
EFN	B9T719	Aspartic proteinase nepenthesin-1, putative	Dioneae, Nepenthes, Cephalotus, Sarracenia	Hatano and Hamada, 2008; Schulze et al., 2012; Lee et al., 2016; Rottloff et al., 2016
EFN	B9RNR8	Aspartic proteinase nepenthesin-2, putative	Dioneae, Nepenthes, Cephalotus, Sarracenia	Hatano and Hamada, 2008; Schulze et al., 2012; Lee et al., 2016; Rottloff et al., 2016
EFN	B9SNP5	Carboxypeptidase	Dioneae	Schulze et al., 2012; Bemm et al., 2016; Lee et al., 2016; Rottloff et al., 2016
EFN	B9S815	Serine carboxypeptidase, putative	Dioneae, Nepenthes	Schulze et al., 2012; Bemm et al., 2016; Lee et al., 2016; Rottloff et al., 2016
EFN	B9T4J8	Xylem serine proteinase 1, putative	–	–
EFN	B9R726	Xylem serine proteinase 1, putative	–	–
FN/EFN	B9RNR9	Aspartic proteinase nepenthesin-2, putative	Dioneae, Nepenthes, Cephalotus	Hatano and Hamada, 2008; Schulze et al., 2012; Lee et al., 2016; Rottloff et al., 2016
FN/EFN	B9T568	Serine carboxypeptidase, putative	Dioneae, Nepenthes	Schulze et al., 2012; Bemm et al., 2016; Lee et al., 2016; Rottloff et al., 2016
**CHITINASES**				
EFN	B9S6S0	Class I chitinase, putative	Dioneae, Nepenthes, Cephalotus, Drosera	Hatano and Hamada, 2012; Schulze et al., 2012; Lee et al., 2016; Rottloff et al., 2016
EFN	B9T8H9	Class IV chitinase, putative	Dioneae, Nepenthes, Cephalotus, Drosera	Hatano and Hamada, 2012; Schulze et al., 2012; Lee et al., 2016; Rottloff et al., 2016
EFN	B9RIP3	Hevamine-A, putative	Dioneae, Nepenthes, Cephalotus, Drosera	Hatano and Hamada, 2012; Schulze et al., 2012; Lee et al., 2016; Rottloff et al., 2016
FN/EFN	B9SBZ8	Chitinase, putative	Dioneae, Nepenthes, Cephalotus, Drosera	Hatano and Hamada, 2012; Schulze et al., 2012; Lee et al., 2016; Rottloff et al., 2016
EFN	B9RIP2	Acidic endochitinase SE2, putative	Dioneae, Nepenthes, Cephalotus, Drosera	Hatano and Hamada, 2012; Schulze et al., 2012; Lee et al., 2016; Rottloff et al., 2016
**LIPASES**				
FN	B9SJ71	Hydrolase, acting on ester bonds, putative (*phospholipase C2*)	Dioneae, Nepenthes	Schulze et al., 2012;
EFN	B9SQQ6	Zinc finger protein, putative (*gdsl esterase/lipase*)	Dioneae, Nepenthes	Schulze et al., 2012; Rottloff et al., 2016
EFN	B9RM21	Zinc finger protein, putative (*gdsl esterase/lipase*)	Dioneae, Nepenthes	Schulze et al., 2012; Rottloff et al., 2016
FN/EFN	B9T8L6	Zinc finger protein, putative (*gdsl esterase/lipase*)	Dioneae, Nepenthes	Schulze et al., 2012; Rottloff et al., 2016
**NUCLEIC ACID HYDROLYSIS**				
FN/EFN	B9SZ66	Wound-induced protein WIN1 (*pathogenesis-related protein 4*)	Dioneae, Nepenthes, Cephalotus	Schulze et al., 2012; Bemm et al., 2016; Lee et al., 2016
FN/EFN	B9SZ67	Wound-induced protein WIN1 (*pathogenesis-related protein 4*)	Dioneae, Nepenthes, Cephalotus	Schulze et al., 2012; Bemm et al., 2016; Lee et al., 2016
**CELL WALL MODIFYING ENZYMES**				
EFN	B9T7M3	Alpha-glucosidase, putative	Nepenthes	Rottloff et al., 2016
EFN	B9RTU8	Basic 7S globulin 2 small subunit (*xylanase inhibitor*)	–	–
EFN	B9RIY8	Beta-glucosidase, putative	Nepenthes	Rottloff et al., 2016
EFN	B9S377	Ceramidase, putative	–	–
EFN	B9RYU9	Endoglucanase	Nepenthes, Dionaea, Cephalotus, Drosera, Sarracenia	Hatano and Hamada, 2012; Schulze et al., 2012; Bemm et al., 2016; Lee et al., 2016
EFN	B9T103	Glucan endo-1,3-beta-glucosidase, putative	Nepenthes, Drosera	Rottloff et al., 2016
EFN	B9SU04	Glucan endo-1,3-beta-glucosidase, putative	Nepenthes, Drosera	Rottloff et al., 2016
EFN	B9SAU3	Pectinesterase	–	–
EFN	B9RD90	Pectinesterase	–	–
EFN	B9RFP1	Polygalacturonase, putative	Dioneae, Nepenthes	Schulze et al., 2012
EFN	B9S447	Putative uncharacterized protein (*xylanase inhibitor*)	–	–
EFN	B9T2C7	Serine-threonine protein kinase (*polygalacturonase inhibitor*)	Dionaea, Nepenthes	Rottloff et al., 2016
FN/EFN	B9T6M9	Glucan endo-1,3-beta-glucosidase, basic isoform, putative	Nepenthes	Rottloff et al., 2016
FN/EFN	B9RBE5	Glucan endo-1,3-beta-glucosidase, putative	Nepenthes	Rottloff et al., 2016
FN/EFN	B9SMA9	Laccase	–	–
FN/EFN	B9RU20	Pectinesterase	–	–
FN/EFN	B9RA18	Pectinesterase	–	–
EFN	B9T3Q0	Non-specific lipid-transfer protein	Dioneae, Nepenthes, Drosera	Schulze et al., 2012; Bemm et al., 2016; Rottloff et al., 2016
EFN	B9SRS0	Non-specific lipid-transfer protein	Dioneae, Nepenthes, Drosera	Schulze et al., 2012; Bemm et al., 2016; Rottloff et al., 2016
EFN	B9SE97	Peroxidase	Dioneae, Nepenthes, Cephalotus	Hatano and Hamada, 2012; Bemm et al., 2016; Lee et al., 2016; Rottloff et al., 2016
EFN	B9R8I7	Multicopper oxidase, putative	–	–
FN/EFN	B9RCG6	Polygalacturonase, putative	Dioneae	Schulze et al., 2012
FN/EFN	B9RJG5	Putative uncharacterized protein (*probable glucan 1,3-beta-glucosidase A*)	Dioneae, Nepenthes	Rottloff et al., 2016
FN/EFN	B9RBC9	Structural constituent of cell wall, putative	–	–
FN	B9SBL2	Multicopper oxidase, putative	–	–
FN/EFN	B9S4B6	Peroxidase	Dionaea, Nepenthes	Hatano and Hamada, 2012; Bemm et al., 2016; Lee et al., 2016; Rottloff et al., 2016
EFN	B9S9S6	Putative uncharacterized protein (*fasciclin-like arabinogalactan protein 1*)	–	–
**FUNCTION IN DEFENSE**				
EFN	B9RC64	Osmotin, putative	Dioneae	Schulze et al., 2012; Bemm et al., 2016; Rottloff et al., 2016
FN/EFN	B9RC65	Osmotin, putative	Dioneae	Schulze et al., 2012; Bemm et al., 2016; Rottloff et al., 2016
EFN	B9T6Y3	Monodehydroascorbate reductase, putative	–	–
EFN	B9REW9	Superoxide dismutase [Cu-Zn]	Dionaea	Schulze et al., 2012
FN/EFN	B9SAZ8	Reticuline oxidase, putative	–	–
EFN	B9SAZ6	Reticuline oxidase, putative	–	–
EFN	B9SB02	Reticuline oxidase, putative	–	–
EFN	B9T0V5	Putative uncharacterized protein (*desiccation-related protein*)	Sarracenia	Fukushima et al., 2017
FN/EFN	B9T0V6	Putative uncharacterized protein (*desiccation-related protein*)	Sarracenia	Fukushima et al., 2017
FN/EFN	B9T346	Carbonic anhydrase, putative	–	–
FN/EFN	B9RC10	Glucose-methanol-choline (Gmc) oxidoreductase, putative	–	–
FN/EFN	B9RGE3	Disease resistance protein RPM1, putative	–	–
EFN	B9S7U9	STS14 protein (*pathogenesis related protein PR-1*)	Dionaea	Schulze et al., 2012
**CARBOHYDRATE METABOLISM**				
FN	B9RG09	Transaldolase, putative		
EFN	B9R9R9	Beta-fructofuranosidase, soluble isoenzyme I, putative	–	–
EFN	B9SRG1	Enolase, putative	Nepenthes	Lee et al., 2016
FN/EFN	B9RAL0	Glyceraldehyde-3-phosphate dehydrogenase	–	–
FN/EFN	B9SP64	Phosphoglucomutase, putative	–	–
**UNKNOWN FUNCTION**				
FN	B9RQ33	5-methyltetrahydropteroyltriglutamate–homocysteine methyltransferase, putative	–	–
FN	B9S0I6	DNA binding protein, putative	–	–
FN	B9SKK5	Nucleoside diphosphate kinase	–	–
FN	B9SCN6	Putative uncharacterized protein	–	–
FN/EFN	B9RWF4	Elongation factor 1-alpha	–	–
EFN	B9RJM9	Putative uncharacterized protein	–	–
EFN	B9RK70	Putative uncharacterized protein	–	–
EFN	B9RNV2	Early nodulin 55-2, putative	–	–
FN/EFN	B9RPP7	DUF26 domain-containing protein 2, putative	–	–
FN/EFN	B9RS28	Mta/sah nucleosidase, putative	–	–
FN/EFN	B9RZI8	Alpha/beta hydrolase, putative	–	–
FN/EFN	B9S225	Mta/sah nucleosidase, putative	–	–
FN/EFN	B9SXP3	Putative uncharacterized protein	–	–
FN/EFN	B9T494	Auxin-induced in root cultures protein 12, putative	–	–
EFN	B9REF0	Hydrolase, hydrolyzing O-glycosyl compounds, putative	–	–

Most of the proteins identified in this study are known to possess defined biochemical activity and/or physiological function in plants. Apart from 15 proteins, the remaining 64 can be tentatively sorted into the seven functional classes as shown in Table 1. Although a sizeable fraction of these proteins was previously identified in EFN and/or FN from other species, enzymes related to the dismantling of the cell wall (four pectinesterases, two polygalacturonases and one polygalacturonase inhibitor), protein hydrolysis (two xylem serine proteinases and one carboxypeptidases) have not been previously identified in any type of nectar.

The limited availability of the complete proteome of nectar from different species precludes a more precise appraisal regarding the distribution of these seven classes of proteins into EFN and FN of other plant taxa. However, as seen in Table 1, 39 out of the 79 proteins listed have identical predicted biochemical activities (biochemical matching partner) in the exudates of five genera of carnivorous plants. Apart from the proteins involved in defense functions, most of the other enzymes display hydrolytic activity against proteins, chitin, carbohydrates, lipids and nucleic acids as well as the capability of hydrolyzing/modifying components of the cell wall of plants or fungi. The presence of these enzymes provides evidence that the EFN and FN possess the enzymatic machinery to promptly digest biological material of microbial, plant or animal origin which happens to land into the floral or extrafloral nectaries.

Floral and extrafloral nectars are thought to possess a set of proteins that constitute the Carter-Thornburg nectar redox cycle, whose concerted action protect the nectar from infection (Liu and Thornburg, 2012). As Table 2 shows, only the EFN has the complete set of proteins of the Carter-Thornburg nectar redox cycle, while in FN only one enzyme (B9SAZ8) from this cycle could be identified, thus probably indicating that FN has alternative modes to avoid microbial infection. Both EFN and FN share a carbonic anhydrase (Table 1), that may act to avoid abrupt changes in the nectar pH, thus stabilizing the different enzymatic activities (see Table 2) in the nectar.

**Table 2:** The Carter-Thornburg redox cycle enzymes identified in the FN and EFN of castor plants (*Ricinus communis*).

EFN	B9REW9	Superoxide dismutase [Cu-Zn]
EFN/FN	B9T346	Carbonic anhydrase, putative
EFN	B9T6Y3	Monodehydroascorbate reductase, putative
EFN	B9RTU8	xyloglucan-specific endo-β-1,4-glucanase inhibitor
EFN	B9S447	xyloglucan-specific endo-β-1,4-glucanase inhibitor
EFN/FN	B9SAZ8	Reticuline oxidase, putative
EFN	B9SAZ6	Reticuline oxidase, putative
EFN	B9SB02	Reticuline oxidase

Thirty proteins were differentially accumulated between EFN and FN (Table S1), and from these, 23 were distributed among all the seven functional classes shown in Table 1, while seven were classified as proteins of unknown function. Of the differentially expressed proteins, a total of 24 were more abundant in the EFN. A desiccation-related protein (B9T0V6) displayed the highest rate of differential expression, followed by a carbonic anhydrase B9T346 and a glucan 1,3-beta-glucosidase (B9RJG5), with a fold change of 17.4, 14.4, and 10.8 respectively. As discussed below, the functional significance of the differential expression of these proteins may bear relation to the persistent nature of the extrafloral nectary as compared to the floral nectary.

## Discussion

We present here a direct comparison between the proteomes of EFN and FN from the same species, collected under similar temporal and environmental conditions. It confirms the greater complexity of the EFN, both in number of proteins species and in terms of biochemical capability, which probably underlies functional differences between the two nectar types.

The mechanisms employed to create an environment hostile to microbial infestation is a moot point in nectar biology (González-Teuber et al., 2009; Park and Thornburg, 2009; Heil, 2015; Roy et al., 2017). The task of creating an environment antagonistic to microbial infestation through the production of hydrogen peroxide seems to be one of the chosen strategies in EFN, as indicated by the presence in its proteome of a full set of proteins from the Carter-Thornburg redox-cycle (Table 2; Carter and Thornburg, 2004). The absence of these proteins in FN shows that rather than to rely on the steady production of hydrogen peroxide, the FN counts with a wide array of hydrolases, which may act in concert to ward off microbial growth. It should also be noted that in EFN the array of hydrolases is wider than in FN, indicating that EFN may rely on at least two different strategies to avoid microbial infection.

One of the most conspicuous evidence for the functional distinction between EFN and FN is the absence in the FN of a beta-fructofuranosidase. This enzyme is known to adjust the carbohydrate composition of the extrafloral nectar to exclude non-mutualistic ants (Heil et al., 2005; González-Teuber et al., 2009). The quantitative analysis we performed (Table S1), provides further support for the biochemical and functional differences between EFN and FN. Most of the differentially expressed proteins were more abundant in the EFN. A desiccation-related protein (B9T0V6), a carbonic anhydrase (B9T346) and a glucan 1, 3-beta-glucosidase (B9RJG5) were the most abundantly expressed. The desiccation-related proteins are involved in promoting the tolerance of plants to desiccation, although an alternative role as defense proteins against microorganisms has been proposed (Zha et al., 2013). Its differential accumulation in the EFN may be causally related to the long period of time in which the extrafloral nectary is metabolically active. The nectar produced at a given period if not consumed is either evaporated or reabsorbed, leading to the periodical increase in the osmotic pressure of the surface of the extrafloral nectary so that the presence of this desiccation related protein would counterbalance the detrimental biological effect of a high osmotic pressure. Carbonic anhydrase is a metalloenzyme that catalyzes the interconversion of CO_2_ and ${\text{HCO}}_{3}^{-}$ and it is suggested to have a role in the stabilization of nectar pH (Park and Thornburg, 2009), thus propitiating the maintenance of the biochemical and functional properties of the nectar proteins; buffering nectar to a physiological pH would be essential if the enzymes in the nectar are to remain active. Finally, glucan 1, 3-beta-glucosidase (B9RJG5) has asserted roles in plant development and hold a well-characterized activity against phytopathogenic fungi (Balasubramanian et al., 2012). Again, the long-lasting nature of the extrafloral nectary as compared to the floral nectary provides a reason for the differential abundance of this protein in the EFN.

The wide array of peptidases, nucleases, lipases, cell wall modifying enzymes and chitinases found in the proteomes of EFN and FN, raises qualms about the contention that action of nectar proteins is limited to prevent microbial growth in the nectar. Apart from few proteins, notably the pectinesterases and polygalacturonases, most of the proteins identified in the proteomes of EFN and FN were previously identified in varied biochemical analysis of nectars from the castor plant and from other sources (see for example: Harper et al., 2010; Orona-Tamayo et al., 2013; Seo et al., 2013; Millán-Cañongo et al., 2014; Zha et al., 2016; Zhou et al., 2016). However, as these studies were generally focused in the identification of proteins that could have a role in maintaining the nectar a pathogen-free environment, the significance of proteins other than the classical pathogen-related proteins, was not reckoned worth of further inquiry. Our proteome analysis support to the idea that one of the roles of the nectar proteins is to prevent microbial growth, keep the nectar pH at the physiological level and provide a pH-balanced meal for visitors (Park and Thornburg, 2009). Although some of the identified proteins are known to be involved in defense reaction, most of the others cannot possibly be involved either in pathogen control or pH maintenance and therefore the adaptive role of these proteins is a question that warrants investigation.

The widely held notion that nectar represents phloem sap, does not find support in the data we present here. The proteomes of FN and EFN have not much in common with the proteomes of phloem, both in terms of number and diversity of functions of the proteins (for reviews see Carella et al., 2016; Rodríguez-Celma et al., 2016). In this context, it is relevant to point out that studies dealing with the transcriptome (Doering-Saad et al., 2006) and proteome (Barnes et al., 2004) of the phloem sap of *R. communis* indicated a much higher number and diversity of proteins than that we found in our proteome analysis of FN and EFN of *R. communis*.

As shown in Table 1, the proteomes of EFN and FN share a number of peptidases, lipases, nucleases, carbohydrases, and chitinases with the exudates of carnivorous plants. These hydrolytic enzymes act to give to the exudates the capability of digesting any prey that happens to be trapped, thus making available to the host plant sources of nitrogen, phosphorous, carbon, etc. (Ellison and Gotelli, 2009; Fukushima et al., 2017; Thorogood et al., 2017). Also shared by EFN, FN and the exudates are the proteins whose activity creates a pathogen-free environment, notably glucanases and chitinases. The identification of pectinesterases and polygalacturonases both in EFN and FN, point out a heightened potential for digesting complex carbohydrates of plant origin, including the major constituents of the cell wall. It thus appears to be likely that any biological material landing in the floral or extrafloral nectaries are liable to be digested, resulting in the production of nitrogen and carbon sources, which may be absorbed by the nectary gland and distributed throughout the plant to provide additional nutrition. This hypothesis begs for a careful experimental testing.

It is usually claimed that carnivory has evolved independently at least six times in five angiosperm orders and seems to be restricted to 0.2% of plant species (Ellison and Gotelli, 2009). However, following the report of a hitherto unknown type of herbivory in underground leaves from three *Philcoxia* species (Pereira et al., 2012), the authors suggested that carnivory may not be a rare trait and that the number of carnivorous plants is underestimated, thus giving support to a notion expressed years before by Chase et al. (2009), which famously claimed that “we are surrounded by murderous plants.” Therefore, whether carnivory is a pervasive trait continues to be a contentious issue, but the common features shown here between the proteomes of EFN and FN of castor plants and the proteome of exudates from carnivorous plants, adds a new twist to this debate: as a result of nectar secretion, extrafloral and floral nectaries are competent to digest biological material from animal or plant origin which land on its surface. Considering that these glands are widespread in the angiosperms and that these proteome features may be shared many other nectars, one is compelled to propose that we are indeed surrounded by “murderous plants.” Whether the hydrolytic capabilities of EFN and FN has any adaptive value and whether the carbon and nitrogen sources generated are absorbed and systemically distributed throughout the plants, are issues entreating cautious experimentation.

Mass spectrometry raw data files are available at: PRIDE Archive (https://www.ebi.ac.uk/pride/archive/) project accession PXD009104.

## Author contributions

FN and FC conceived and designed research. FN, AF, and FT conducted sample preparation and proteomics experiments. FN, AF, GD, and FC analyzed data. FN, AF, GD, and FC wrote the manuscript. All authors read and approved the manuscript.

### Conflict of interest statement

The authors declare that the research was conducted in the absence of any commercial or financial relationships that could be construed as a potential conflict of interest.
